# Physiological changes in captive elephants in northern Thailand as a result of the COVID-19 tourism ban—stress biomarkers

**DOI:** 10.3389/fvets.2024.1351361

**Published:** 2024-02-09

**Authors:** Jarawee Supanta, Janine L. Brown, Pakkanut Bansiddhi, Chatchote Thitaram, Veerasak Punyapornwithaya, Khanittha Punturee, Patcharapa Towiboon, Nopphamas Somboon, Jaruwan Khonmee

**Affiliations:** ^1^Faculty of Veterinary Medicine, Chiang Mai University, Chiang Mai, Thailand; ^2^Center of Elephant and Wildlife Health, Chiang Mai University Animal Hospital, Chiang Mai University, Chiang Mai, Thailand; ^3^Elephant, Wildlife, and Companion Animals Research Group, Chiang Mai University, Chiang Mai, Thailand; ^4^Smithsonian Conservation Biology Institute, Center for Species Survival, Front Royal, VA, United States; ^5^Department of Medical Technology, Faculty of Associated Medical Sciences, Chiang Mai University, Chiang Mai, Thailand; ^6^Small Animal Hospital, Faculty of Veterinary Medicine, Chiang Mai University, Chiang Mai, Thailand

**Keywords:** Asian elephant, welfare, glucocorticoids, stress leukogram, oxidative stress, malondialdehyde, 8-hydroxy-2′-deoxyguanosine

## Abstract

The international travel ban instituted by the Thai government in March 2020 in response to the COVID-19 pandemic greatly affected how tourist camp elephants were managed, with reductions in exercise opportunities, longer chaining hours, and diminished food provisioning. This study was conducted to determine how those changes affected health and welfare biomarkers in individual elephants over the 2 years of the countrywide lockdown (April 2020–April 2022). Blood and fecal samples were collected from 58 elephants at six camps (monthly in Year 1, quarterly in Year 2) and analyzed for stress biomarkers — fecal glucocorticoid metabolites (fGCM), serum oxidative stress [malondialdehyde (MDA) and 8-hydroxy-2′-deoxyguanosine (8-OHdG)], and stress leukograms. Overall, fGCM concentrations increased within the first few months and remained higher than pre-COVID levels, as did the H/L ratio, a measure affected by cortisol. Serum 8-OHdG, an indicator of DNA oxidative damage, also increased over time, while monocytosis and lymphopenia further suggested alterations in immune function as a result of stress. By contrast, another marker of oxidative stress, serum MDA, declined, possibly in response to reduced roughage and supplement intake. A notable finding was a seasonal pattern of fGCM that was significantly different from previous studies. Whereas higher fGCM during the rainy season were observed in this study, previously, concentrations were highest during the winter, high tourist season. Thus, ironically, both the presence and absence of tourists have been associated with increased fGCM concentrations, albeit for different reasons. Camp management factors negatively affecting stress outcomes included shorter chain lengths, longer chain hours, lack of exercise, and reduced roughage and supplements. Overall, it was clear that camps struggled to maintain adequate care for elephants during the COVID-19 pandemic, highlighting the importance of tourist income and need for contingency plans to cope with potential future disruptions to tourism.

## Introduction

The Asian elephant (*Elephas maximus*) is classified as endangered on the International Union for Conservation of Nature (IUCN) Red List of Threatened Species™ (2016) and listed under Appendix I by the Convention on International Trade in Endangered Species of Wild Fauna and Flora (1). In Thailand, the elephant is revered as a national symbol and plays a crucial role in Thai cultural traditions and Buddhist practices, while also significantly contributing to the country’s economic landscape. Presently, Thailand is home to approximately 3,500 captive elephants, with the majority (~95%) under private ownership and engaged primarily in tourism (2). Most (~60%) are in the northern and northeastern regions of the country, predominantly in Chiang Mai province (3). Tourist activities vary and include hands-off opportunities like observation from afar or walking alongside to more interactive activities like feeding, bathing, riding with a saddle or bareback, and elephant shows (4). Our previous studies of how tourism affects elephant health and welfare found that feeding high energy foods (banana and sugar cane) was associated with obesity and alterations in lipid profiles and metabolic function (insulin, glucose, fructosamine, glucose to insulin ratio), while fecal glucocorticoid metabolite (fGCM) concentrations as a proxy for stress were actually lower in riding elephants in relation to more exercise and better body condition (5–7), similar to findings in U.S. zoos (8–10). Poor foot scores were associated with longer work hours and walking distances, and being on concrete, while skin wounds were related to improper restrain equipment used by mahouts (e.g., ankus/bullhook, chains) and higher fGCM (11). Thus, while some tourist activities may be a benefit to elephant health (5–7), others can contribute to poor welfare through long work hours, misuse of the ankus, stress associated with being too close to tourists, and harsh training to allow hands-on interactions (11, 12). Based on these empirical findings, a set of welfare guidelines was developed for the Asian Elephant Specialist Group (13) and adapted by Captive Elephant Standards^1^ that conducts on-demand welfare audits of elephant camps throughout southeast Asia. As a result, improvements in tourist camp management based on these research findings were beginning to take hold (14). Then the pandemic hit.

When SARS-CoV-2, the virus that causes COVID-19, was recognized by Thailand in March 2020 (12), the tourism landscape changed drastically. The Thai government banned all international travel, severely reducing foreign tourism and associated income (15). Consequently, all tourist camps closed, leading to further concerns over welfare of elephants and mahouts (elephant keepers). In a previous study, surveys were conducted throughout the first 2 years of the country-wide lockdown (15), with data compared to before COVID-19 (4) and identified significant changes in camp structure and elephant management. Elephant activities, distance walked, and amounts of food were reduced, while chain hours were increased. Thus, effects on animal health were expected. Indeed, changes in several health biomarkers were observed, including lower serum creatine kinase and increased liver enzyme, AST and ALP, concentrations possibly due to physical inactivity or altered nutrition. By contrast, reduced feeding of bananas and sugar cane was associated with lower body condition scores (BCS), less obesity, and lower TG concentrations (16) suggesting some physical improvements. This companion study further examines patterns of several stress biomarkers throughout the 2-year COVID-19 tourism ban to examine effects on health and welfare.

Exposure to harmful stimuli over time can lead to adverse effects on animal well-being (17), generally through activation of the hypothalamo-pituitary–adrenal (HPA) axis and resulting secretion of glucocorticoids (GC) from the adrenal cortex (18). Downstream effects of increased GC production include suppression of immune function (19, 20), reproduction (21–23), increased disease susceptibility (19, 24, 25), and altered carbohydrate metabolism (26, 27). Hematology changes also can occur with stress, including an increase in the neutrophil to lymphocyte (N/L) ratio (28, 29). Neutrophils (or heterophils, depending on the species) and lymphocytes are involved in immunological processes and constitute almost 80% of all white blood cells in mammals (30). Interestingly, in elephants and other *Afrotheria* like hyraxes and manatees, heterophils are produced instead of neutrophils (31). Thus, in those species, heterophil to lymphocyte (H/L) ratios are calculated instead. Leukocyte numbers react to stress, but in different directions: e.g., heterophils increase, while lymphocyte numbers decrease during a stressful event (32, 33). Studies further show that an increase in GC can drive changes in heterophil and lymphocyte numbers (32–34). Indeed, one study found a positive relationship between the H/L ratio and fecal GC concentrations (fGCM) in logging Asian elephants in Myanmar, irrespective of sex, age, or environmental context (35). Stress leukograms vary among species but are typically characterized by lymphopenia and neutrophilia, with occasional monocytosis and eosinopenia (36). Serum malondialdehyde (MDA), a byproduct of lipid peroxidation also has been associated with high levels of GC hormones (37, 38). An increase in free radicals causes overproduction of MDA, so it is commonly used as a marker of oxidative stress and antioxidant status (39) and could be measured in addition to GCs to assess welfare in working elephants. Finally, 8-hydroxy-2′-deoxyguanosine (8-OHdG) is a biomarker of DNA oxidative damage and formed when oxygen radicals damage DNA. It can be measured in blood, as well as noninvasively in urine and feces, and so has become another potential marker of welfare (40–43).

Given that changes in metabolic health were observed in tourist elephants during the COVID-induced travel ban (16), the goals of this study were to evaluate how stress biomarkers (GCs, serum MDA, serum 8-OHdG, stress leukograms, and H/L ratio) were affected in these same elephants. As in the previous study, some biomarker and BCS data were compared to earlier studies on tourist elephants before the pandemic (5–7, 12, 34, 44–47). Ultimately, it is hoped that this information will be used to develop management plans that can ensure good welfare of elephants with or without tourists, and contribute to strategies that improve overall health, welfare and population sustainability within the tourism paradigm.

## Materials and methods

### Human and animal ethics statements

The research received approval from the Human Research Ethics Committee (HS1/2564) and the Institutional Animal Care and Use Committee at the Faculty of Veterinary Medicine (FVM), Chiang Mai University (CMU), located in Chiang Mai, Thailand (FVM-ACUC; S4/2564).

### Study population

A total of 58 adult elephants (14 males and 44 females) from six elephant camps in the Mae Taeng district of Chiang Mai province was studied and initially described by Supanta et al. (15, 16) (Table 1). Preceding the nationwide lockdown, elephants participated in five tourist-related activities: riding with a saddle, riding bareback, elephant shows, feeding, bathing or observation only. As a result of the lockdown, all tourist-related activities ceased. The inclusion criteria for elephants in this study were: adult age (18–50 years); deemed healthy by camp veterinarians or veterinary consultants; females were not pregnant. Samples were not collected from males during musth for safety reasons. These are the same elephants included in the two companion papers (15, 16).

**Table 1:** Summary (mean ± SEM, range) of tourist, elephant and mahout numbers, and elephant management at six Thailand tourist camps during the COVID-19 pandemic and international travel ban between April 2020 and April 2022.

Parameters	Time	Camp A	Camp B	Camp C	Camp D	Camp E	Camp F
Participating elephant age (years)		32.2 ± 2.7	37.9 ± 2.9	35.4 ± 3.3	40.3 ± 2.6	45.2 ± 2.8	43.3 ± 6.7
		(19.0–50.0)	(22.0–56.0)	(20.0–56.0)	(28.0–54.0)	(40.0–56.0)	(30.0–50.0)
Mahout age (years)		35.7 ± 2.2	35.7 ± 1.8	42.3 ± 3.2	30.9 ± 2.0	44.2 ± 3.4	25.7 ± 4.7
		(25.0–48.0)	(23.0–47.0)	(18.0–58.0)	(23.0–40.0)	(36.0–51.0)	(20.0–35.0)
Tourist number/day	T00 (Before COVID-19)	200	600	300	600	50	15
	T01 (April 2020)	0	4	2	4	4	0
	T04 (January–April 2021)	0	4	2	4	4	0
	T07 (January–April 2022)	6	10	6	10	10	7
Mahout number/camp	T00 (Before COVID-19)	40	66	66	12	10	3
	T01 (Apr 2020)	40	54	66	12	10	3
	T04 (January–April 2021)	20	28	39	7	5	3
	T07 (January–April 2022)	18	20	25	4	4	2
Elephant number/camp^a^	T00 (Before COVID-19)	13:30	18:36	26:41	0:12	0:12	0:3
	T01 (April 2020)	13:30	18:36	26:41	0:12	0:12	0:3
	T04 (January–April 2021)	13:41	18:31	26:34	0:8	0:7	0:3
	T07 (January–April 2022)	13:35	18:28	12:28	0:5	0:7	0:3
Participating elephants^a^	T01 (April 2020)	5:9	4:10	5:7	0:10	0:5	0:3
	T04 (January–April 2021)	5:9	2:10	4:7	0:8	0:5	0:3
	T07 (January–April 2022)	2:8	0:7	1:4	0:6	0:5	0:3
Chain time^b^ (h/day)	T00 (Before COVID-19)	16.0	16.0	16.0	16.0	17.0	18.0
	T01 (April 2020)	22.5	22.0	23.5	16.0	17.0	20.0
	T04 (January–April 2021)	22.6 ± 0.1	48.0	23.5	48.0	14.6 ± 0.2	20.0
		(22.5–23.3)				(14.0–15.0)	
	T07 (January–April 2022)	22.7 ± 0.2	24.0	23.5	24.0	14.6 ± 0.2	20.0
		(22.5–24.0)				(14.0–15.0)	
Chain length (m)	T00 (Before COVID-19)	2.0	3.0	1.5	3.0	5.0	3.0
	T01 (April 2020)	3.9 ± 0.7	3.0	1.5	3.0	1.2 ± 0.1	3.0
		(1.5–10.0)				(1.0–1.5)	
	T04 (January–April 2021)	4.3 ± 0.8	3.0	1.5	3.0	1.2 ± 0.1	2.0
		(1.5–10.0)				(1.0–1.5)	
	T07 (January–April 2022)	5.1 ± 1.0	3.0	2.5	3.0	1.2 ± 0.1	2.0
		(1.5–10.0)				(1.0–1.5)	
Walking distance (km/day)	T00 (Before COVID-19)	6.0	10.0	20.0	6.0	2.0	3.0
	T01 (April 2020)	1.5	5.6 ± 0.0	4.0	1.0	1.0	3.0
			(0.0–6.0)				
	T04 (January–April 2021)	1.4 ± 0.1	2.0	1.3 ± 0.0	0.5	0.5	3.0
		(0.8–1.5)		(1.2–1.3)			
	T07 (January–April 2022)	1.1 ± 0.2	2.0	2.8 ± 0.8	1.0	2.0	3.0
		(0.0–1.5)		(2.0–6.0)			
Amount of roughage (kg/day)	T00 (Before COVID-19)	200.0	120.0	180.0	120.0	180.0	300.0
	T01 (April 2020)	192.3 ± 2.8	139.1 ± 4.6	180.0	120.0	180.0	200.0
		(180.0–200.0)	(120.0–150.0)				
	T04 (January–April 2021)	190.0 ± 3.8	134.1 ± 6.8	150.0	120.0	180.0	150.0
		(165.0–200.0)	(100.0–150.0)				
	T07 (January–April 2022)	154.6 ± 3.1	131.8 ± 7.6	120.0	120.0	180.0	150.0
		(150.0–180.0)	(100.0–150.0)				
Amount of supplement (kg/day)	T00 (Before COVID-19)	20.0	25.0	25.0	25.0	25.0	15.0
	T01 (April 2020)	14.2 ± 2.1	25.0	25.0	25.0	15.0	20.0
		(5.0–20.0)					
	T04 (January–April 2021)	4.7 ± 0.2	8.3 ± 1.2	1.0	10.0	15.0	5.0
		(3.0–5.0)	(1.0–10.0)				
	T07 (January–April 2022)	10.2 ± 1.8	11.9 ± 2.1	10.0	10.0	15.0	5.0
		(1.0–15.0)	(1.0–15.0)				

a Number of males:females.

b Elephants were chained for 48 consecutive hours.

Yearly data are compared to pre-COVID-19 (T00) as described in Supanta et al. (15).

### Data collection

Data collection for this prospective study began in April 2020 (T01), a month after the implementation of a travel ban due to the COVID-19 pandemic, and continued through April 2022 (T07) as camps began to open again to tourists. The study consisted of in-person interviews with camp owners, veterinarians and/or mahouts (elephant keepers) to gather information about the numbers of tourists, elephants, and mahouts, tourist activities (riding, walking, shows, feeding), chaining duration and length, and provisioning of roughage and supplements (14). The questionnaire is shown in Table 1. Interviews were conducted, and samples were collected monthly for the first year [April 2020 to April 2021 (T01-T04)] and then every 4 months during the second year [May 2021 to April 2022 (T05-T07)]. This methodology led to a total of 16 on-site visits that were subsequently segmented into seven time periods for analysis [April 2020 (T01), May–August 2020 (T02), September–December 2020 (T03), January–April 2021 (T04), May–August 2021 (T05), September–December 2021 (T06), January–April 2022 (T07)] as described previously (15, 16). Queries about camp management before COVID-19 were included in the first interview to capture data on operations in 2019 (16). A total of seven camp managers, 50 mahouts, and two veterinarians were interviewed across the 16 data collection periods. Supplementary information on camp management and elephant activities pre-COVID-19 was sourced from Bansiddhi et al. (4, 11, 12) and Norkaew et al. (5–7).

### Fecal collection

Fecal samples were collected monthly in the morning between 08.00 to 12.00 h to avoid variation in the circadian rhythm of cortisol secretion (23, 48, 49). Defecation was either observed or indicated to have occurred within the hour by the mahout. Fecal boluses were broken open and approximately 50 grams of material taken from the middle and placed into zip lock plastic bags labeled with the elephant name, time of collection and date. Samples were transported at 2–8°C in a cool box to the Faculty of Veterinary Medicine, Chiang Mai University and then stored at −20°C until fecal extraction and hormonal analysis.

### Blood collection

Blood (~8 mL) was collected from a caudal auricular vein and placed into two tubes: serum separator tubes (SST) (~7 mL) for analysis of oxidative stress (MDA, 8-OHdG), and ethylenediaminetetraacetic acid tubes (EDTA) (~1 mL) for the stress leukogram (heterophils, lymphocytes, monocytes and eosinophils). Blood in SST tubes was centrifuged at 1,200×*g* for 15 min within 6 h of collection (50), while whole EDTA blood was analyzed within 24 h of blood smear preparation. Serum was stored at −20°C until processing and analysis.

### Fecal extraction and glucocorticoid analysis

Fecal samples were extracted as described by Norkaew et al. (5). Frozen feces were thawed at room temperature (RT) and dried in a conventional oven (60°C) for 24–48 h. Mixed powdered feces (~0.1 g) were placed into glass tubes, 4.5 mL of EtOH and 0.5 mL of distilled water was added, and tubes were vortexed briefly. Samples were extracted by boiling in a water bath (90°C) for 20 min, with 95% EtOH added to keep the volume at 5 mL, and then centrifuged at 1,200×*g* for 20 min. The fecal extracts were combined, dried in a 90°C water bath, re-suspended in 3 mL of 95% EtOH, dried down again, and finally re-suspended in 1 mL of 50% methanol. Samples were stored at −20°C until analysis.

Concentrations of fGCM were measured in extracts diluted 1:3 in assay buffer (0.0137 M Trizma base, 0.2 M Tris–HCl, 0.2 M NaCl, 0.2 M EDTA, 0.001% BSA, and 0.001% Tween 20; pH 7.5) using a double-antibody EIA with a polyclonal rabbit anti-corticosterone antibody (CJM006, Coralie Munro, UC Davis, CA) previously used in Thailand (5). Samples and corticosterone standards (50 μL) were added to wells in duplicate followed by corticosterone-HRP (25 μL; 1:30,000) and anti-corticosterone antibody (25 μL, 1:100,000). Plates were incubated in the dark at RT for 2 h, followed by 100 μL of TMB solution and incubation for 20–35 min. Stop solution was added (50 μL, 1 N HCl) and absorbance measured at 450 nm (TECAN, Männedorf, Switzerland). Assay sensitivity was 0.12 ng/mL. Intra- (at 90% binding) and inter-assay coefficients of variation (CV, based on dose values) were < 10% (all duplicates over 10% were reanalyzed) and 12.28%, respectively.

### Oxidative stress

MDA was quantified by a thiobarbituric acid (TBA)-reacting substances (TBARS) assay described previously (51, 52). Briefly, 50 μL of serum samples and standards were mixed with 750 μL of 0.44 M phosphoric acid, 250 μL of 42 mM TBA and 450 μL of distilled water. Tubes were placed into a boiling water bath for 15 min, cooled on ice for 5 min, and then centrifuged at 900×*g* for 5 min. Absorbance of the supernatant was measured at 532 nm using a UV–VIS spectrophotometer (Shimadzu, Japan). Serum MDA concentrations were calculated from standard curves of MDA equivalents generated by the acid-catalyzed hydrolysis of 1,1,3,3-tetramethoxypropane (TMP) within the range of 1.25–80.00 μM. Assay sensitivity was 0.15 μM. Intra- and inter-assay coefficients of variation (CV) were 1.1 and 2.3%, respectively.

Serum 8-OHdG was measured using a DetectX^®^ DNA Damage Enzyme Immunoassay Kit (Cat #K059-H5, Arbor Assays) validated for Asian elephants (52, 53). Assay sensitivity was 0.072 ng/mL and intra-assay and inter-assay CVs were < 10 and 6.3%, respectively.

### Stress leukogram

White blood cell count differentials were done manually on blood smears stained with Wright–Giemsa (RVL Supply, Pathumthani, Thailand) (45). A ratio of heterophils to lymphocytes was calculated for each sample.

### Statistical analyses

Elephant data from management surveys are presented as mean ± standard error of the mean (SEM) and a range depending on the type of data. Statistical analyses were conducted using R version 4.3.1 (54) package geepack, function geeglm, under the optimal autoregressive order 1 correlation structure (55). Repeated measures data were analyzed using Generalized Estimating Equations (GEE) to determine the effect of camp management practices on stress parameters. A univariate analysis was performed, and any variable with a significance level of *p* < 0.15 was included in the subsequent multivariate analysis. *Post hoc* tests were conducted using Tukey’s multiple comparisons of least-squares mean (LS-mean) to identify differences between variable categories. For the multivariate model, Sex and Age were incorporated as covariate factors. The selection of the final model in the GEE analysis was determined based on the smallest quasi-likelihood under the independence model criterion (QIC) values (56) using package MuMIn, function dredge (57) with the significance set at α = 0.05. Reference categories were chosen based on specific questions as described previously (11, 12), including Sex = male; Time = T01 (April 2020, the beginning of the travel ban); and Camp = F (the smallest camp). Differences in mean fGCM, oxidative stress markers, stress leukograms and H/L ratio among three seasons (summer: February 16–May 15; rainy: May 16–October 15; winter: October 16–February 15) (53, 58, 59) were analyzed by Tukey’s post-hoc tests following GEE analyses.

## Results

### Descriptive statistics of camp management variables

Camp differences in management practices before the COVID-19 travel ban (T00) compared to the start of the lockdown (T01) and each subsequent year (T04, T07) are described in Supanta et al. (15) and Table 1. A summary of Time and Camp differences in biomarkers of adrenal activity, oxidative stress, DNA damage, stress leukogram, and the heterophil/lymphocyte ratio are shown in Supplementary Table S1. Univariate and multivariate GEE results are presented in Supplementary Tables S1–S9. Table 2 summarizes the results of multivariate GEE analyses of category and continuous camp management variables associated with stress biomarkers, with LS-mean data from the final GEE models presented in Table 3. Stress biomarkers were compared to published reference ranges before COVID-19 in Table 4. Moreover, seasonal effects are presented in Supplementary Table S10.

**Table 2:** Summary of significant relationships from multivariate GEE analyses of category and continuous camp management variables associated with stress biomarkers.

Parameters	Category camp management variables			Continuous camp management variables					
	Sex	Time	Camp	Age	Walking distance (km/day)	Chain length (m)	Chain hour (h/day)	Roughage (kg/day)	Supplement (kg/day)
**Glucocorticoids**									
Fecal glucocorticoid metabolites (ng/g)	NS	S	S	NS	NS	NS	NS	NS	NS
**Oxidative stress**									
Malondialdehyde (μM)	NS	S	NS	NS	NS	S (−)	S (+)	S (+)	NS
8-hydroxy-2′-deoxyguanosine (ng/ml)	NS	S	S	NS	NS	S (+)	S (−)	NS	NS
**Stress leukogram**									
Heterophils (×10^3^ cells/μL)	NS	NS	NS	NS	NS	NS	NS	S (+)	NS
Monocytes (×10^3^ cells/μL)	NS	S	S	S (−)	NS	NS	NS	NS	NS
Lymphocytes (×10^3^ cells/μL)	NS	S	NS	S (−)	NS	NS	S (+)	NS	NS
Eosinophils (×10^3^ cells/μL)	NS	NS	S	S (−)	NS	NS	NS	S (+)	NS
Heterophil/Lymphocyte ratio	NS	S	S	S (+)	S (+)	NS	NS	NS	NS

NS, no significance; S, significance; (+) positive relationship; (−) negative relationship.

**Table 3:** Mean (± SEM) and range values for biomarkers of adrenal activity, oxidative stress, DNA damage, stress leukogram, and the heterophil/lymphocyte ratio in captive Asian elephants (*n* = 58) in six Thailand tourist camps during the COVID-19 pandemic and international travel ban between April 2020 and April 2022.

	Before COVID-19			During COVID-19		
Parameters	Mean ± SEM	Min–Max	Mean range	Mean ± SEM	Min–Max	Mean range
**Glucocorticoids**						
Fecal glucocorticoid metabolites (ng/g)	50.8 ± 0.9^3^ 53.5 ± 0.7^4^ 33.9 ± 1.2^5^	11.4–194.2^3^ 8.2–291.6^4^ 21.7–75.5^5^	34.5–72.3^3^ 33.2–67.4^4^ ND	55.8 ± 17.2^10^ 52.7 ± 0.9^11^	22.2–106.1^10^ 16.9–239.7^11^	40.3–76.5^11^
**Oxidative stress**						
Malondialdehyde (μM)	3.3 ± 0.1^8^	2.5–4.4^8^	ND	1.4 ± 0.4^9^ 1.7 ± 0.3^10^ 2.2 ± 0.0^11^	0.9–2.5^9^ 1.0–3.0^10^ 0.3–6.1^11^	1.6–2.6^11^
8-hydroxy-2’-deoxyguanosine (ng/mL)	ND	ND	ND	7.3 ± 1.9^10^ 7.5 ± 0.1^11^	3.7–15.2^10^ 3.3–18.2^11^	6.9–8.8^11^
**Stress leukogram**						
Heterophils (× 10^3^ cells/μL)	ND	ND	0.8–13.5^2^	3.0 ± 0.1^11^	0.8–15.6^11^	2.0–4.0^1^
Monocytes (× 10^3^ cells/μL)	ND	ND	0.0–3.3^2^	3.6 ± 0.1^11^	0.0–10.2^11^	2.0–4.0^1^
Lymphocytes (× 10^3^ cells/μL)	ND	ND	1.7–12.0^2^	4.4 ± 0.1^11^	0.1–22.1^11^	0.5–0.8^1^
Eosinophils (× 10^3^ cells/μL)	ND	ND	0.0–1.2^2^	0.5 ± 0.1^11^	0.0–7.7^11^	0.01–1.0^1^
Heterophil/Lymphocyte ratio	ND	0.3–2.8^6^ 0.2–3.5^7^	ND	0.8 ± 0.3^11^	0.1–1.8^11^	0.5–1.4^11^

ND, Not determined.

1 Mikota (44).

2 Janyamethakul et al. (45).

3 Norkaew et al. (5).

4 Bansiddhi et al. (12).

5 Kosaruk et al. (46).

6 Seltmann et al. (34).

7 Seltmann et al. (35).

8 Khonmee et al. (60).

9 Kosaruk et al. (52).

10 Kosaruk et al. (53).

11 Results from the current study.

### Fecal glucocorticoid metabolites

**Figure 1:**
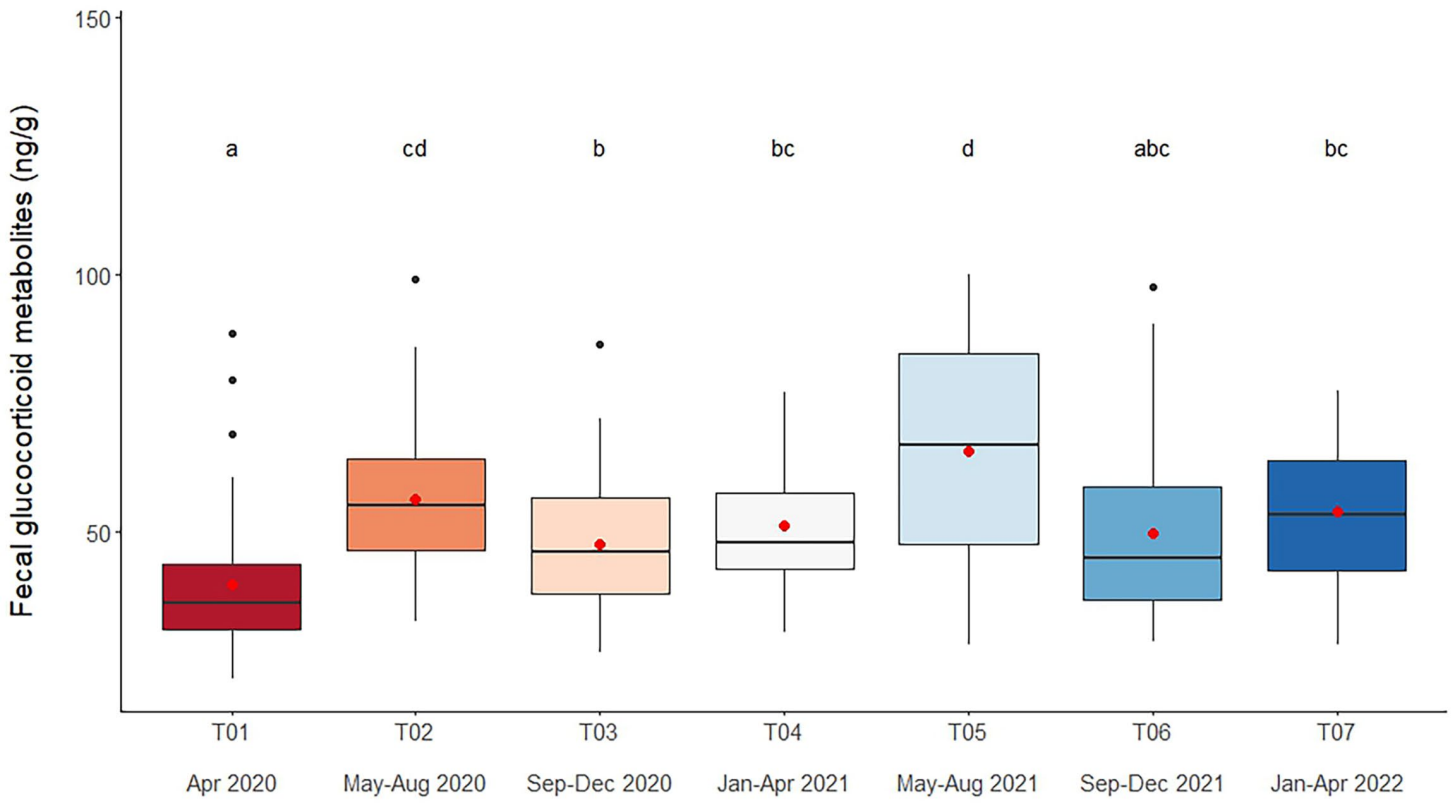
Boxplots of fecal glucocorticoid metabolite concentrations in captive Asian elephants (*n* = 58) over a 2-year period during the COVID-19 pandemic and international travel ban. Each plot shows the median (horizontal line), mean (red dot), 25 and 75% quartile ranges, and minimum and maximum ranges (whiskers). Different letters indicate significant differences between time periods (*p* < 0.001).

Overall average fGCM concentration was 52.9 ± 1.1 ng/g (21.2–147.4 ng/g), with lower overall concentrations observed in T01 (*p* < 0.001) (Figure 1 and Table 4). Variables significantly affecting fGCM concentrations in the multivariate model were Time and Camp (Tables 2, 4 and Supplementary Table S2), with a rising trend in three of six camps (Camps A, C, E) when data were compared across T01, T04 and T07 time periods (Supplementary Table S1). Elephants at Camps B and F exhibited the highest fGCM concentrations, while those at Camp C had the lowest overall. There was a seasonal effect on fGCM, with the lowest concentrations in the summer and highest values in the rainy season (Supplementary Table S10).

**Table 4:** LS-mean (± SEM) glucocorticoids, oxidative stress, stress leukogram and heterophil/lymphocyte ratio of Asian elephants (*n* = 58) in relation to categorical demographic and management variables from final GEE models.

Variable		*N*	fGCM (ng/g)	MDA (μM)	8-OHdG (ng/mL)	Heterophils (×10^3^ cells/μL)	Monocytes (×10^3^ cells/μL)	Lymphocytes (×10^3^ cells/μL)	Eosinophils (×10^3^ cells/μL)	H/L
**Hypothesis** ^**1**^			**+**	**+**	**+**	**+**	**+**	**−**	**−**	**+**
**Time**										
	T01	50	41.6 ± 2.3^a^	2.7 ± 0.1^c^	7.2 ± 0.3^a^	3.0 ± 0.1^a^	3.5 ± 0.2^ab^	4.6 ± 0.1^bc^	0.4 ± 0.0^a^	0.7 ± 0.0^a^
	T02	51	56.3 ± 1.9^cd^	2.2 ± 0.1^ab^	7.5 ± 0.3^ab^	2.9 ± 0.1^a^	3.1 ± 0.1^a^	4.8 ± 0.1^c^	0.4 ± 0.0^a^	0.7 ± 0.0^a^
	T03	50	47.9 ± 1.6^ab^	2.1 ± 0.1^a^	7.5 ± 0.3^ab^	2.9 ± 0.1^a^	3.7 ± 0.1^b^	4.5 ± 0.1^bc^	0.3 ± 0.0^a^	0.8 ± 0.1^ab^
	T04	45	52.4 ± 1.9^bc^	2.0 ± 0.1^a^	7.2 ± 0.3^a^	2.9 ± 0.1^a^	3.6 ± 0.2^ab^	3.7 ± 0.1^a^	0.3 ± 0.0^a^	0.8 ± 0.1^ab^
	T05	40	65.9 ± 3.2^d^	2.2 ± 0.1^ab^	8.2 ± 0.4^ab^	2.8 ± 0.2^a^	3.8 ± 0.2^ab^	3.7 ± 0.2^a^	0.3 ± 0.0^a^	0.8 ± 0.1^ab^
	T06	38	49.1 ± 2.7^abc^	2.4 ± 0.1^b^	8.2 ± 0.4^ab^	2.8 ± 0.1^a^	4.0 ± 0.2^b^	3.9 ± 0.2^ab^	0.3 ± 0.1^a^	0.8 ± 0.1^ab^
	T07	36	53.3 ± 2.0^bc^	2.0 ± 0.1^a^	9.0 ± 0.4^b^	2.9 ± 0.2^a^	4.1 ± 0.3^b^	3.1 ± 0.2^a^	0.4 ± 0.0^a^	1.0 ± 0.1^b^
**Camp**										
	A	13	56.8 ± 1.8^c^	2.3 ± 0.2^a^	7.8 ± 0.3^ab^	3.0 ± 0.1^a^	4.1 ± 0.2^b^	4.0 ± 0.1^a^	0.5 ± 0.0^cd^	0.8 ± 0.0^bc^
	B	9	56.3 ± 2.5^c^	2.3 ± 0.1^a^	6.8 ± 0.2^a^	2.7 ± 0.1^a^	3.3 ± 0.1^a^	4.1 ± 0.1^ab^	0.3 ± 0.0^bc^	0.7 ± 0.0^ab^
	C	8	41.8 ± 1.2^a^	2.3 ± 0.1^a^	6.9 ± 0.2^a^	2.6 ± 0.1^a^	3.8 ± 0.2^ab^	4.6 ± 0.1^b^	0.2 ± 0.0^a^	0.6 ± 0.0^a^
	D	8	53.7 ± 2.1^bc^	2.1 ± 0.1^a^	7.9 ± 0.5^ab^	2.9 ± 0.1^a^	3.8 ± 0.2^ab^	4.0 ± 0.2^ab^	0.3 ± 0.0^bc^	0.8 ± 0.1^bc^
	E	5	46.9 ± 1.7^ab^	2.3 ± 0.1^a^	8.1 ± 0.3^ab^	3.1 ± 0.2^a^	3.9 ± 0.3^ab^	3.7 ± 0.2^a^	0.4 ± 0.0^bcd^	0.9 ± 0.1^c^
	F	3	58.6 ± 2.8^c^	2.2 ± 0.1^a^	9.4 ± 0.5^b^	3.2 ± 0.3^a^	3.3 ± 0.2^a^	3.9 ± 0.3^ab^	0.6 ± 0.1^d^	0.8 ± 0.1^bc^
**Sex**										
	Male	14	50.8 ± 2.5^a^	2.3 ± 0.1^a^	7.1 ± 0.2^a^	2.8 ± 0.1^a^	3.6 ± 0.2^a^	4.5 ± 0.2^a^	0.4 ± 0.0^a^	0.8 ± 0.1^a^
	Female	45	52.2 ± 1.1^a^	2.2 ± 0.0^a^	7.6 ± 0.2^b^	2.9 ± 0.1^a^	3.7 ± 0.1^a^	4.1 ± 0.1^a^	0.3 ± 0.0^a^	0.8 ± 0.0^a^

^a,b,c^Different letters across columns indicate significant differences for each variable (*p* < 0.001).

1 Predicted change over Time categories.

fGCM, fecal glucocorticoid metabolites; MDA, malondialdehyde; 8-OHdG, 8-hydroxy-2′-deoxyguanosine; H/L, heterophil/lymphocyte ratio.

### Oxidative stress

**Figure 2:**
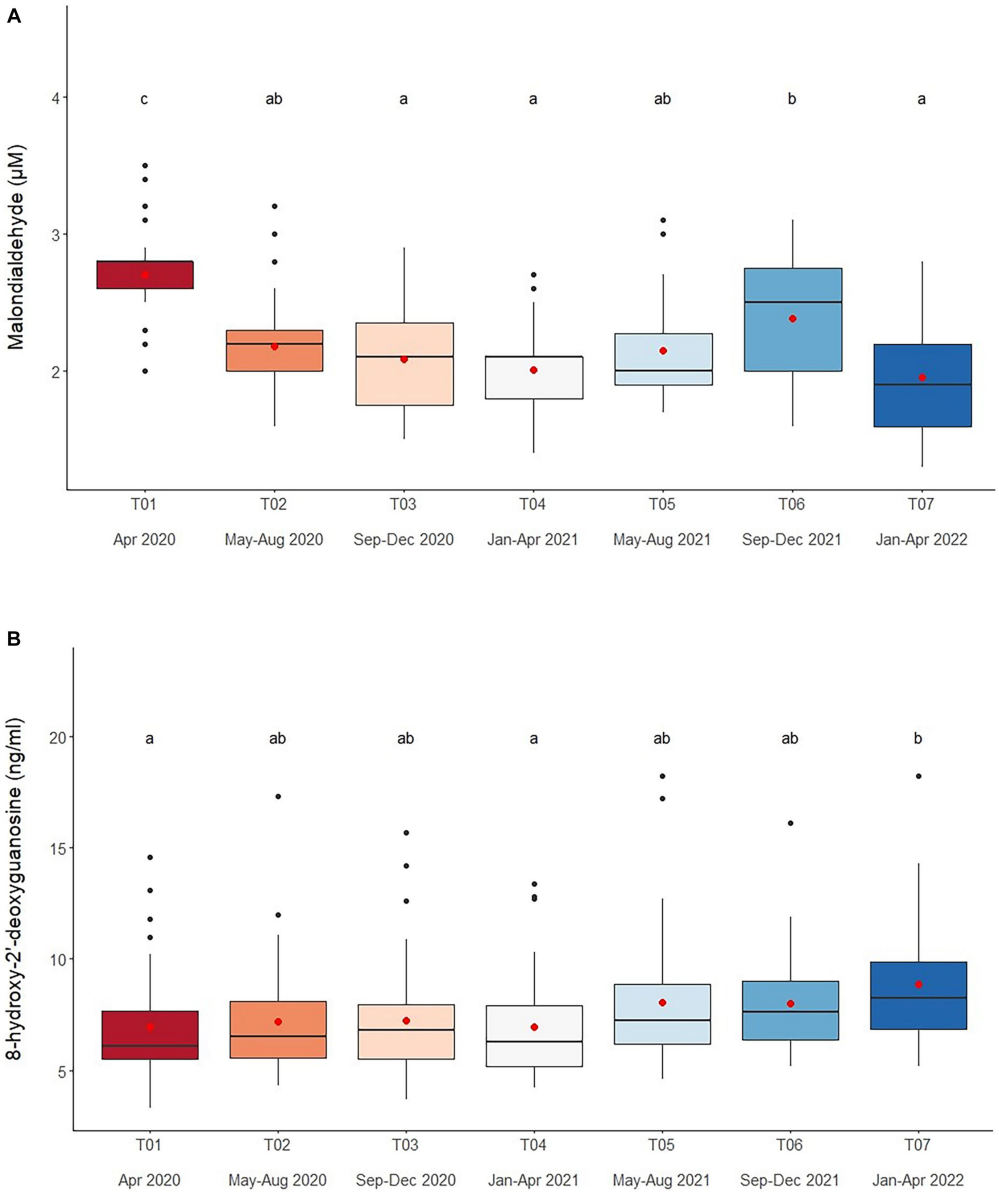
Boxplots of oxidative stress markers **(A)** malondialdehyde and **(B)** 8-hydroxy-2′-deoxyguanosine in captive Asian elephants (*n* = 58) over a 2-year period during the COVID-19 pandemic and international travel ban. Each plot shows the median (horizontal line), mean (red dot), 25 and 75% quartile ranges, and minimum and maximum ranges (whiskers). Different letters indicate significant differences between time periods (*p* < 0.001).

Serum MDA concentrations averaged 2.2 ± 0.5 μM (1.3–3.8 μM), and were affected by Time, Chain length, Chain hour and Roughage in the multivariate analysis (Tables 2, 4 and Supplementary Table S3). Chain hour and Roughage were positive relationships, while Chain hour was negative (Table 1 and Supplementary Table S3). There was a declining trend over time (Figure 2A and Table 4) in all six camps (Supplementary Table S1), but no seasonal effect (Supplementary Table S10).

Overall average serum 8-OHdG concentration was 7.5 ± 1.4 mg/mL (3.3–18.2 mg/mL) and related to Time, Camp, Chain length and Chain hour in the final multivariable model (Tables 2, 4 and Supplementary Table S4). Chain length was positively associated with serum 8-OHdG concentration, while a negative relationship with Chain hour approached significance (Table 2 and Supplementary Table S4). There was an increasing trend over the 2-year period (Figure 2B and Table 4), albeit significant in only two camps (Camp C and E) (Supplementary Table S1). Overall, Camp F had consistently higher 8-OHdG values, while Camp B had among the lowest concentrations. There was no seasonal effect on serum 8-OHdG concentrations (Supplementary Table S10).

### Stress leukogram

**Figure 3:**
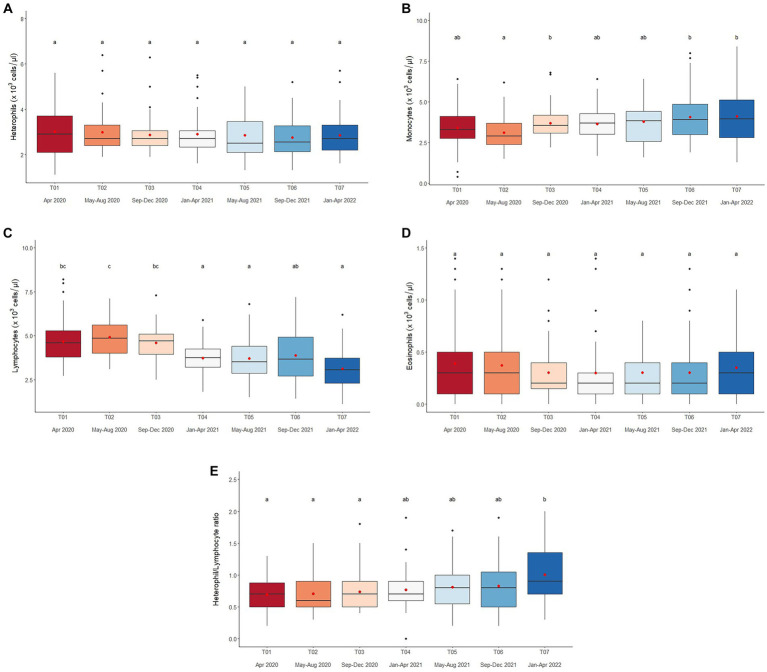
Boxplots of white blood cell counts in the stress leukogram for **(A)** heterophils, **(B)** monocytes, **(C)** lymphocytes, **(D)** eosinophils, and the **(E)** heterophil/lymphocyte ratio in captive Asian elephants (*n* = 58) over a 2-year period during the COVID-19 pandemic and international travel ban. Each plot shows the median (horizontal line), mean (red dot), 25 and 75% quartile ranges, and minimum and maximum ranges (whiskers). Different letters indicate significant differences between time periods (*p* < 0.001).

Overall average heterophil counts were 2.9 ± 0.1 × 10^3^ cells/μL (1.1–8.9 × 10^3^ cells/μL) and affected only by Roughage (positive) in the multivariate analysis (Tables 2, 4 and Supplementary Table S5). There were no differences among Time periods (Figure 3A and Table 4), and counts were not affected by season (Supplementary Table S10).

Monocyte counts averaged 3.7 ± 0.1 × 10^3^ cells/μL (0.0–8.7 × 10^3^ cells/μL). From the multivariate analysis, factors that had the greatest influence were Age, Time, and Camp (Tables 2, 4 and Supplementary Table S6). Monocyte counts were higher in younger elephants (Table 1 and Supplementary Table S6) and exhibited an increasing trend over the 2-year period (Figure 3B and Table 4). At each time period, Camp A elephants consistently had lower monocyte counts (Supplementary Table S1). There was no seasonal effect (Supplementary Table S10).

Mean lymphocyte counts overall were 4.2 ± 0.1 × 10^3^ cells/μL (1.1–9.9 × 10^3^ cells/μL). From the multivariate analysis, Age, Time, and Chain hour had the greatest influence (Tables 2, 4 and Supplementary Table S7), with a negative relationship to Age and positive relationship to Chain hour (Table 2 and Supplementary Table S7). An overall declining trend over time was observed (Figure 3C and Table 2) that was significant in four camps (Camps A, C, E, F) (Supplementary Table S1). There was no effect of season (Supplementary Table S10).

Eosinophil counts averaged 0.3 ± 0.0 × 10^3^ cells/μL (0.0–1.7 × 10^3^ cells/μL), and from the multivariate analysis, Age, Time, Camp and Roughage had significant effects. Eosinophil counts were lower in older elephants, and there was a positive relationship with Roughage (Table 2 and Supplementary Table S8). However, changes over time revealed no discernable pattern (Figure 3D and Table 4), and neither were their seasonal effects (Supplementary Table S10). Elephants at Camp C had the lowest eosinophil counts, while those at Camp F were generally the highest (Table 4).

For the overall H/L ratio (0.8 ± 0.01; range, 0.2–3.4) Age, Time, Camp and Walking distance had significant effects. H/L ratio was higher in older elephants, with a positive relationship to Walking distance (Table 2 and Supplementary Table S9) and increasing trend over the 2-year period (Figure 3E and Table 4) for four camps (Camps A, C, E, F) (Supplementary Table S1). Camp E had the highest H/L ratio, while Camp C was the lowest. There were no seasonal effects (Supplementary Table S10).

## Discussion

After the closure of Thailand’s borders in response to the COVID-19 pandemic in March 2020, a study was undertaken to determine the impact of the COVID-19 pandemic-induced international travel ban on the management, health, and welfare of captive elephants. In the first paper, we describe marked changes in physical activity and access to nourishment, with heightened durations of chaining, and decreases in numbers of mahouts as camps closed due to the lack of tourists (15). We then showed how significant alterations in muscle and liver enzymes, lipid profiles, and body condition occurred in relation to these management changes, indicating potential adverse effects on elephant health (16). The current investigation provides additional evidence of physiological shifts based on measures of stress biomarkers in these same elephants, specifically those associated with adrenal GC activity, oxidative stress, and hematology, that suggests these biological changes may be caused by chronic stress. Overall, concentrations of fGCM and serum 8-OHdG increased, as did the H/L ratio, and remained elevated throughout the travel ban, potentially in relation to prolonged inactivity. By contrast, MDA concentrations decreased and remained low, possibly as a result of dietary modifications. Based on hematology, there was evidence of monocytosis and lymphopenia, possibly due to stress-related increases in corticosteroids. Camp management factors negatively affecting stress outcomes included shorter chain lengths, longer chain hours, lack of exercise, and restricted food resources. Altogether, these studies exposed the struggles that camps experienced in trying to maintain adequate care for elephants in the absence of tourists and associated income, and the need for contingency plans to better prepare for any future disruptions to tourism.

### Fecal glucocorticoid metabolites

Concentrations of fGCM increased over time and were highest at T05 (May–August 2021), a year into the lockdown. The range in this study (16.9–239.7 ng/g) aligns with previous values in Asian elephants in northern Thailand [37.4–98.3 ng/g (5–7); 39.2–69.0 ng/g/feces (12); 25.8–39.3 ng/g (46); 22.2–106.1 ng/g (53)], albeit at the higher end as the travel ban progressed. While there are limited studies on how tourist or logging activities impact stress hormone responses, there are indications that yearly patterns of GCs may be related more to work activities than environmental influences (52, 53). In India, fGCM concentrations were higher in elephants involved in an intensive and crowded public festival, but not other tourist activities (61). In Myanmar, elephants have the best body condition, but also experience the highest fGCM concentrations as they enter the more stressful logging and monsoon seasons (62). By contrast, a different study of the Myanmar population found the highest fGCM concentrations in the winter and lowest in the monsoon season, with intermediate values in the summer (63). That study was conducted on non-working elephants only; thus, it appears to demonstrate a true seasonal pattern.

In Thailand, a series of correlational studies aimed at identifying captive management factors that exert significant influences on welfare outcomes have been conducted by our group over the past 6 years (4–7, 11, 12, 14–16, 45–47, 49, 52, 53, 64). Lower fGCM concentrations were found to be associated with riding or walking activities, being kept in open areas during the day, and in the forest or under a tree at night (12). High BCSs were linked to limited exercise and feeding of high-calorie treats like banana and sugarcane, and also higher fGCM concentrations (5–7); all these were similar to findings observed in U.S. zoo elephants (8, 9). However, the relationship between fGCM and BCS during COVID-19 differed, as there was a slight, but significant negative relationship between fGCM and BCS (Spearman’s rank correlation; rho = −0.14; *p* = 0.0172). So, as body condition decreased, presumably due to limited roughage and supplements, fGCM increased. The reason for this is not clear, as there were no clear relationships between fGCM and management factors found to be important in earlier studies, like Walking distance, Chain hours, Chain length, Roughage or Supplements. Rather it could simply be due to the fact that elephants during the COVID-19 pandemic were chained most, if not all, of the time and had nothing to do, and body condition was reduced to levels not seen in earlier studies. In a study of wild elephants in India, fGCM were highest in individuals with the lowest BCSs, lower than those observed in this study (65). So, it might not be surprising there were little differences among camps in elephant adrenal responses to management, as it was all inadequate. Follow up studies are now planned as tourism begins to return normal in Thailand, although as of the end of 2023 it had not.

A significant new finding was related to season, where the pattern in this study was opposite to that found earlier. Previously, higher fGCM concentrations during the winter months suggested a possible link between colder temperatures and increased adrenal activity (5, 6, 46). However, those months (November–February) also correspond to the high tourist season (5, 6), so until now, it has been impossible to tease apart tourist activity versus climate conditions on adrenal activity. But this and another study (53) conducted during the COVID-19 lockdown strongly suggest that tourist activities are the most likely cause of increased fGCM excretion during the winter, high tourist season months. Thus, further studies are needed to better understand not only what tourist activities affect elephant health and welfare, but how. That said, it is critical to consider context, as increases in adrenal GC activity can occur in response to both positive and negative stimuli, so higher concentrations do not necessarily equate to poor welfare, nor do lower levels indicate good welfare (66).

### Oxidative stress

Serum MDA concentrations (1.3–3.8 μM) were similar to previous ranges [0.93–3.0 μM (44, 45)], but showed a declining trend as the study progressed, with T01 (April 2020) being the highest and T07 (January–April 2020) the lowest. There was no obvious seasonal effect, which agrees with another study conducted during the same time period in Thai elephants (53), although a follow-up study did find somewhat lower concentrations during the rainy season (52). Polyunsaturated fatty acids are major substrates for lipid peroxidation (59, 61, 62), and MDA is an end-product produced by the decomposition of the lipid peroxidation process (61). We recently found that elephants during COVID-19 received reduced high-energy diets from a lack of tourists (16), resulting in lower lipid concentrations and BCSs (16). Thus, one possible explanation is a reduction in fat consumption due to dietary changes, as the factor, Roughage was positively associated with MDA concentrations and generally contains 4.1–5.5% crude fat (64). During COVID-19, amounts of roughage decreased, leading to possible reductions in lipid intake. Moreover, roughage, especially young Napier grass, has antioxidant properties (67) known to inhibit the activity of free radicals (68, 69), which might also explain the observed lower MDA concentrations during the lockdown.

Several studies have suggested monitoring serum 8-OHdG as an indicator of DNA damage might be a useful biomarker for both acute (70, 71) and chronic stress (72). In this study, serum 8-OHdG concentrations (3.7–15.2 ng/mL) were similar to a previous study in elephants (3.3–18.2 ng/mL, (52, 53), but with an increasing trend over time, being lowest at the beginning and highest at the end of the study. A previous study found inactive mice had higher oxidative stress markers (lipid hydroperoxides, vascular superoxide release and nicotinamide adenine dinucleotide phosphate oxidase) than active mice (42). In humans, positive correlations have been observed between urinary 8-OHdG and average working hours and serum cortisol concentrations (69), suggesting that increased GC activity may lead to higher levels of oxidative DNA damage (37). In zoo-housed grizzly bears (*Ursus arctos horribilis*), fecal 8-OHdG concentrations were positively related to fGCM concentrations (71). Thus, it is possible that the increased GCs noted in this study may have contributed to increased DNA damage (73), although other factors may certainly have been involved.

### Stress leukogram and H/L ratio

In human and animal medicine, a stress leukogram is characterized by neutrophilia, lymphopenia, eosinopenia, and monocytosis, and specifically refers to the effects of corticosteroids on white blood cell populations (36). While the hematology values during COVID-19 were generally within the range of healthy elephants (45, 74, 75), comparatively, data point to the presence of monocytosis and lymphopenia, two of four criteria in a stress leukogram. Monocytosis was most likely the result of a shift from a marginalized to a circulating population of monocytes. Changes in the expression of adhesion molecules and chemotactic cytokines that interfere with the trafficking of leukocytes into tissues (76) could play a role in changes in WBC populations. Lymphopenia is the result of an immediate shift of lymphocytes from the circulating blood to other tissues, as well as decreased lymphopoeisis (77). In addition, GCs can induce apoptosis of lymphoid cells in mammals (78) and thus interfere with immune functionality. Although no elephants became visibly sick during the study, we cannot rule out subclinical changes as a result of long-term stress associated with restrictive management changes.

The H/L values in this study ranged from 0.1 to 2.5, which agrees with earlier reports of 0.30 to 2.8 (34) and 0.2 to 3.5 (63) in captive Asian timber elephants. The H/L ratio reflects the ability of an animal to cope with infection through injury (via heterophils) or disease (via lymphocytes) (79). While heterophils form the first line of immune defense against bacterial pathogens in inflammatory lesions, lymphocytes are involved in host defense through cytokine production and immune responses (80). As early as 1989, the H/L ratio has been used as an indicator of welfare and how stress affects immune function in response to adrenal-corticoid hyperactivity (81, 82), with lower ratios suggesting better immune function or as a proxy of lower GC responses. During the travel ban, H/L ratios increased over time, which agrees with concomitant increases in GCs that are known to affect the H/L. In elephants, Ukonaho et al. (63) found elephants in Myanmar had higher fGCM concentrations and H/L ratios during the cold season, indicating increased stress. Heterophilia occurs only under stressful conditions, similar to the situation with monocytes, but in this study was not enough to be statistically different. In relation to management factors, there was a positive correlation between lymphocyte numbers and Chain Time, which is consistent with a report showing captive rodents presented higher lymphocyte counts than free-ranging ones (83). Thus, results further suggest elephants in Thailand were exposed to chronic stress during the COVID-19 lockdown.

## Conclusion

This and our two companion studies (15, 16) are the first to reveal how a disruption to the tourist industry had significant negative effects on captive elephant health and welfare in Thailand. Throughout the 2-year international travel ban, there were clear effects of management changes because of the lack of tourists on several stress biomarkers reflecting adrenal activity, oxidative stress, and leukograms. Serum MDA as a measure of oxidative stress declined during the COVID-19 countrywide lockdown in relation to dietary changes. Serum 8-OHdG concentrations were increased, suggesting stressors (via glucocorticoid activity) can physically damage DNA. Higher H/L ratio, along with monocytosis and lymphopenia were two criteria of the stress leukogram that appear to reflect increased glucocorticoid activity due to physical inactivity. One aim was to identify what camps adapted to still meet elephant welfare needs in the absence of tourists that could serve as models for other camps. However, only Camp C had lower stress biomarker profiles, including fGCM, serum 8-OHdG, monocyte counts, and H/L ratios, potentially suggesting elephants were less stressed. Still, those elephants were chained all but 30 min a day, with short chain lengths of 2.5 m and < 3 km/d of walking time - not drastically different from the other camps (40). Thus, it was strikingly clear that camps struggled to care for elephants during the COVID-19 lockdown when there was little incentive to provide exercise or other activities, including socialization, to the elephants. It is hoped that elephant owners will recognize the seriousness of these inadequacies and develop contingency plans to avoid such problems in the future should other events cause disruptions to the tourist industry.

## Data availability statement

The original contributions presented in the study are included in the article/Supplementary material, further inquiries can be directed to the corresponding author.

## Ethics statement

The research received approval from the Human Research Ethics Committee (HS1/2564) and the Institutional Animal Care and Use Committee at the Faculty of Veterinary Medicine (FVM), Chiang Mai University (CMU), located in Chiang Mai, Thailand (FVM-ACUC; S4/2564). The study was conducted in accordance with the local legislation and institutional requirements.

## Author contributions

JS: Conceptualization, Formal analysis, Investigation, Methodology, Validation, Writing – review & editing, Visualization, Writing – original draft. JB: Formal analysis, Methodology, Validation, Writing – review & editing, Conceptualization, Funding acquisition, Resources. PB: Conceptualization, Formal analysis, Investigation, Methodology, Writing – review & editing. CT: Conceptualization, Funding acquisition, Resources, Writing – review & editing. VP: Methodology, Software, Validation, Writing – review & editing. KP: Methodology, Validation, Writing – review & editing. PT: Methodology, Validation, Writing – review & editing. NS: Methodology, Validation, Writing – review & editing. JK: Conceptualization, Data curation, Formal analysis, Funding acquisition, Investigation, Methodology, Resource, Project administration, Supervision, Validation, Visualization, Writing original draft, Writing – review & editing.

## Footnotes

^1^ https://www.elephantstandards.com
