# Supplementary material for: Physiological changes in captive elephants in northern Thailand as a result of the COVID-19 tourism ban—stress biomarkers
Source: Front Vet Sci. 2024 Feb 9;11:1351361. doi: 10.3389/fvets.2024.1351361 (PMC10884277; doi:10.3389/fvets.2024.1351361)
Supplement: Supplementary file 1 [file Data_Sheet_1.pdf]

Supplementary Material

Physiological Changes in Captive Elephants in Northern Thailand as a Result of the COVID-19 Tourism Ban – Stress biomarkers

Jarawee Supanta[1,2], Janine L. Brown[2,3,4†], Pakkanut Bansiddhi[1,2,3], Chatchote Thitaram[1,2,3], Veerasak Punyapornwithaya[1], Khanittha Punturee[5], Patcharapa Towiboon[2], Nopphamas Somboon[6], and Jaruwan Khonmee[1,2,3†*]

*** Correspondence:** Jaruwan Khonmee: jaruwan.khonmee@cmu.ac.th

1 Supplementary Data

The raw measurements are provided in:

- Questionnaire on camp management variables

2 Supplementary Tables

2.1 Supplementary Tables

Supplementary Table 1. Time and camp differences (mean ± SEM, range) in biomarkers of adrenal activity, oxidative stress, DNA damage, stress leukogram, and the heterophil/lymphocyte ratio in captive Asian elephants (n = 58) in six Thailand tourist camps during the COVID-19 pandemic and international travel ban between April 2020 – April 2022.

Parameters	Time	Camp A	Camp B	Camp C	Camp D	Camp E	Camp F
**Glucocorticoids**							
Fecal glucocorticoid metabolites (ng/g)	T01 (Apr 2020)	40.9 ± 2.5$^{b, x}$ (30.9-56.3)	44.4 ± 6.5$^{ab, x}$ (25.9-88.6)	29.0 ± 1.3$^{a, x}$ (21.2-36.4)	42.1 ± 4.2$^{b, x}$ (31.3-60.5)	37.5 ± 2.4$^{b, x}$ (28.8-44.8)	55.9 ± 9.6$^{ab, x}$ (43.4-79.4)
	T04 (Jan-Apr 2021)	55.8 ± 3.7$^{bc, xy}$ (35.8-76.9)	61.4 ± 3.4$^{c, x}$ (45.4-74.2)	51.0 ± 4.0$^{bc, y}$ (39.7-78.7)	48.7 ± 2.1$^{b, x}$ (35.9-56.2)	38.2 ± 2.7$^{a, x}$ (30.2-47.1)	57.9 ± 7.2$^{bc, x}$ (40.4-68.8)
	T07 (Jan-Apr 2022)	57.2 ± 3.5$^{ab, y}$ (41.9-75.6)	61.2 ± 3.8$^{b, x}$ (48.7-77.4)	44.7 ± 4.0$^{a, y}$ (37.8-62.6)	46.5 ± 5.7$^{ab, x}$ (28.0-64.4)	55.0 ± 4.1$^{ab, y}$ (45.3-66.3)	52.6 ± 6.1$^{ab, x}$ (40.4-64.6)
**Oxidative stress**							
Malondialdehyde (µM)	T01 (Apr 2020)	2.8 ± 0.1$^{a, y}$ (2.5-3.8)	2.7 ± 0.1$^{a, y}$ (2.0-3.4)	2.8 ± 0.1$^{a, y}$ (2.5-3.8)	2.6 ± 0.1$^{a, y}$ (2.0-3.2)	2.8 ± 0.2$^{a, y}$ (2.2-3.5)	2.6 ± 0.2$^{a, y}$ (2.3-3.1)
	T04 (Jan-Apr 2021)	2.1 ± 0.1$^{a, x}$ (1.5-2.7)	2.1 ± 0.1$^{a, x}$ (1.8-2.4)	2.1 ± 0.1$^{a, x}$ (1.5-2.7)	1.8 ± 0.1$^{a, x}$ (1.4-2.4)	2.1 ± 1.4$^{a, x}$ (1.8-2.6)	2.0 ± 0.2$^{a, x}$ (1.5-2.3)
	T07 (Jan-Apr 2022)	1.9 ± 0.1$^{ab, x}$ (1.4-2.5)	2.2 ± 0.1$^{b, xy}$ (1.9-2.8)	1.8 ± 0.1$^{a, x}$ (1.4-2.0)	1.8 ± 0.2$^{ab, x}$ (1.6-1.7)	2.2 ± 0.2$^{ab, x}$ (1.6-2.5)	1.8 ± 0.4$^{ab, xy}$ (1.3-2.8)
8-hydroxy-2'-deoxyguanosine (ng/ml)	T01 (Apr 2020)	6.6 ± 0.6$^{ab, x}$ (3.3-11.8)	6.0 ± 0.4$^{a, x}$ (4.4-11.0)	6.5 ± 0.5$^{ab, x}$ (4.4-7.0)	7.6 ± 1.1$^{ab, x}$ (5.6-14.6)	7.7 ± 0.5$^{ab, x}$ (5.7-9.0)	10.3 ± 1.3$^{b, x}$ (7.5-13.1)
	T04 (Jan-Apr 2021)	6.7 ± 0.6$^{a, x}$ (4.6-12.8)	6.4 ± 0.5$^{a, x}$ (4.6-9.5)	6.2 ± 0.5$^{a, x}$ (4.6-8.4)	7.7 ± 1.3$^{a, x}$ (4.2-13.4)	8.1 ± 0.8$^{a, xy}$ (5.9-10.3)	8.3 ± 0.8$^{a, x}$ (6.7-10.1)
	T07 (Jan-Apr 2022)	9.9 ± 1.0$^{a, x}$ (6.7-18.2)	7.6 ± 0.6$^{a, x}$ (5.7-11.2)	8.7 ± 0.6$^{a, y}$ (6.9-10.7)	8.4 ± 1.4$^{a, x}$ (5.2-14.3)	10.3 ± 0.7$^{a, y}$ (8.1-12.1)	10.2 ± 1.4$^{a, x}$ (7.6-13.3)
**Stress leukogram**							
Heterophils (x 10^3 cells/µl)	T01 (Apr 2020)	4.0 ± 0.2$^{c, y}$ (2.7-5.6)	2.7 ± 0.2$^{b, x}$ (1.6-4.3)	2.8 ± 0.3$^{ab, x}$ (1.1-5.4)	2.6 ± 0.2$^{b, x}$ (1.7-3.7)	3.3 ± 0.5$^{bc, x}$ (2.2-7.7)	1.9 ± 0.1$^{a, x}$ (1.8-2.1)
	T04 (Jan-Apr 2021)	2.6 ± 0.1$^{a, x}$ (2.0-2.9)	2.5 ± 0.2$^{a, x}$ (1.6-3.3)	2.7 ± 0.2$^{a, x}$ (1.9-4.4)	4.7 ± 0.6$^{b, x}$ (2.8-8.9)	3.2 ± 0.5$^{ab, x}$ (2.2-5.4)	3.4 ± 0.4$^{ab, y}$ (2.5-3.9)
	T07 (Jan-Apr 2022)	3.0 ± 0.2$^{a, x}$ (1.6-4.1)	3.1 ± 0.5$^{a, x}$ (1.7-5.2)	2.6 ± 0.2$^{a, x}$ (2.0-3.5)	2.4 ± 0.2$^{a, x}$ (1.9-3.3)	3.3 ± 0.6$^{a, x}$ (1.9-5.7)	2.6 ± 0.3$^{a, x}$ (1.9-3.3)
Monocytes (x 10^3 cells/µl)	T01 (Apr 2020)	3.7 ± 0.3$^{a, x}$ (2.3-8.7)	3.0 ± 0.3$^{a, x}$ (0.4-4.9)	3.8 ± 0.3$^{a, x}$ (1.6-5.7)	3.7 ± 0.3$^{a, x}$ (2.7-7.7)	3.1 ± 0.4$^{a, x}$ (1.3-4.1)	2.2 ± 0.6$^{a, x}$ (0.7-3.1)
	T04 (Jan-Apr 2021)	3.6 ± 0.3$^{ab, x}$ (2.0-5.5)	2.6 ± 0.3$^{a, x}$ (0.0-3.9)	4.0 ± 0.3$^{b, x}$ (2.4-5.8)	4.0 ± 0.3$^{b, x}$ (2.6-5.6)	4.1 ± 0.6$^{ab, x}$ (2.5-6.4)	4.0 ± 0.3$^{b, y}$ (3.5-4.8)
	T07 (Jan-Apr 2022)	3.9 ± 0.4$^{a, x}$ (2.3-6.3)	3.7 ± 0.6$^{a, x}$ (1.3-5.6)	4.4 ± 0.8$^{a, x}$ (2.2-7.1)	4.2 ± 0.6$^{a, x}$ (2.3-6.5)	5.0 ± 1.0$^{a, x}$ (2.4-8.4)	4.0 ± 0.2$^{a, y}$ (3.6-4.3)
Lymphocytes (x 10^3 cells/µl)	T01 (Apr 2020)	4.6 ± 0.3$^{ab, y}$ (3.2-6.8)	4.2 ± 0.3$^{ab, x}$ (2.7-5.6)	5.8 ± 0.4$^{b, y}$ (4.1-8.2)	4.4 ± 0.4$^{ab, x}$ (2.7-5.3)	4.6 ± 0.5$^{ab, y}$ (3.4-6.6)	3.7 ± 0.8$^{a, xy}$ (2.7-5.6)
	T04 (Jan-Apr 2021)	4.1 ± 0.2$^{b, y}$ (2.8-5.9)	3.0 ± 0.2$^{a, x}$ (1.8-4.0)	3.8 ± 0.2$^{ab, y}$ (2.7-4.9)	3.0 ± 0.3$^{a, x}$ (1.9-4.3)	3.9 ± 0.5$^{ab, y}$ (2.4-5.1)	3.8 ± 0.2$^{ab, y}$ (3.4-4.3)
	T07	2.9 ± 0.2$^{a, x}$	4.0 ± 0.6$^{a, x}$	3.4 ± 0.4$^{a, x}$	3.0 ± 0.4$^{a, x}$	2.6 ± 0.4$^{a, x}$	2.4 ± 0.1$^{a, x}$

	(Jan-Apr 2022)	(1.5-4.4)	(1.9-6.2)	(2.2-4.2)	(1.1-4.2)	(1.6-3.9)	(2.3-2.5)
Eosinophils (x 10^3 cells/µl)	T01 (Apr 2020)	0.5 ± 0.1$^{b, x}$ (0.1-1.4)	0.1 ± 0.0$^{a, x}$ (0.1-1.0)	0.1 ± 0.0$^{a, x}$ (0.0-0.4)	0.2 ± 0.1$^{ab, x}$ (2.7-7.7)	0.3 ± 0.0$^{b, x}$ (0.1-1.2)	0.6 ± 0.3$^{ab, x}$ (0.3-1.3)
	T04 (Jan-Apr 2021)	0.5 ± 0.1$^{b, x}$ (0.1-1.3)	0.1 ± 0.0$^{a, y}$ (0.1-0.2)	0.1 ± 0.0$^{a, x}$ (0.1-0.4)	0.2 ± 0.1$^{ab, x}$ (0.1-0.6)	0.3 ± 0.0$^{b, x}$ (0.2-0.5)	0.6 ± 0.3$^{ab, x}$ (0.2-1.4)
	T07 (Jan-Apr 2022)	0.4 ± 0.1$^{a, x}$ (0.1-0.8)	0.4 ± 0.1$^{a, y}$ (0.1-0.8)	0.2 ± 0.1$^{a, x}$ (0.1-0.5)	0.3 ± 0.1$^{a, x}$ (0.1-0.4)	0.3 ± 0.1$^{a, x}$ (0.1-0.7)	0.5 ± 0.3$^{a, x}$ (0.1-1.1)
Heterophil/Lymphocyte ratio	T01 (Apr 2020)	0.9 ± 0.1$^{b, xy}$ (0.6-1.3)	0.7 ± 0.1$^{ab, x}$ (0.3-1.2)	0.5 ± 0.1$^{a, x}$ (0.2-1.3)	0.6 ± 0.1$^{ab, x}$ (0.3-1.0)	0.9 ± 0.1$^{ab, xy}$ (0.5-1.2)	0.6 ± 0.1$^{ab, x}$ (0.3-0.7)
	T04 (Jan-Apr 2021)	0.6 ± 0.1$^{a, x}$ (0.1-0.9)	0.9 ± 0.1$^{a, x}$ (0.5-1.5)	0.7 ± 0.1$^{a, y}$ (0.4-1.0)	1.4 ± 0.4$^{a, x}$ (0.5-3.4)	0.8 ± 0.1$^{a, x}$ (0.6-1.1)	1.0 ± 0.2$^{a, xy}$ (0.7-1.4)
	T07 (Jan-Apr 2022)	1.1 ± 0.1$^{ab, y}$ (0.5-1.7)	0.7 ± 0.1$^{a, x}$ (0.3-2.5)	0.8 ± 0.1$^{ab, y}$ (0.6-1.0)	1.0 ± 0.2$^{ab, x}$ (0.5-2.0)	1.3 ± 0.2$^{b, y}$ (0.7-1.7)	1.1 ± 0.1$^{ab, y}$ (0.8-1.4)

^{a,b,c,d}Values in the same row are significantly different across camps (P <0.001).

^{x,y,z}Values in the same column are significantly different across time periods (P <0.001).

Supplementary Table 2. Univariate and multivariate GEE analyses of demographic and camp management variables associated with fecal glucocorticoid metabolite concentrations.

Variable		N	Univariate analysis			Multivariate analysis		
			Estimate	SE	P value	Estimate	SE	P value
**Sex**								
	Male	14	Reference					
	Female	45	1.470	2.686	0.585			
**Age**			0.011	0.098	0.910			
**Time**								
	T01	51	Reference					
	T02	53	14.92	3.140	<0.001	14.722	2.923	<0.001
	T03	50	6.340	3.030	0.036	4.819	2.968	0.104
	T04	46	11.090	3.310	<0.001	10.015	3.099	0.001
	T05	41	25.54	4.360	<0.001	22.427	4.155	<0.001
	T06	39	8.460	3.780	0.025	4.967	4.009	0.215
	T07	37	12.540	3.230	<0.001	9.502	3.449	0.006
**Camp**								
	A	13	-2.110	3.900	0.589	-0.129	3.432	0.970
	B	9	-2.570	4.060	0.527	-3.787	3.819	0.321
	C	8	-18.040	3.560	<0.001	-17.647	3.047	<0.001
	D	8	-5.060	4.060	0.213	-8.6812	4.238	0.041
	E	5	-12.230	3.890	0.002	-11.628	3.397	<0.001
	F	3	Reference					
**Walking distance (km/day)**			0.408	0.706	0.560			
**Chain length (m)**			1.296	0.644	0.044	-0.561	0.564	0.320
**Chain hour (h/day)**			0.064	0.134	0.630			
**Roughage (kg/day)**			-0.083	0.037	0.025	-0.099	0.053	0.062
**Supplement (kg/day)**			-0.077	0.143	0.590			

SE = Standard error

Variables having a P value <0.15 at the univariate analysis were included in the multivariate analysis.

Supplementary Table 3. Univariate and multivariate GEE analyses of demographic and camp management variables associated with malondialdehyde concentrations.

**Variable**		**N**	**Univariate analysis**			**Multivariate analysis**		
			**Estimate**	**SE**	**P value**	**Estimate**	**SE**	**P value**
**Sex**								
	Male	14	Reference					
	Female	45	-0.059	0.073	0.420			
**Age**			0.002	0.003	0.440			
**Time**								
	T01	51	Reference					
	T02	53	-0.562	0.069	<0.001	-0.565	0.068	<0.001
	T03	50	-0.656	0.070	<0.001	-0.559	0.087	<0.001
	T04	46	-0.736	0.067	<0.001	-0.671	0.088	<0.001
	T05	41	-0.598	0.0749	<0.001	-0.604	0.093	<0.001
	T06	39	-0.317	0.098	0.001	-0.292	0.105	0.006
	T07	37	-0.792	0.087	<0.001	-0.679	0.093	<0.001
**Camp**								
	A	13	0.071	0.121	0.560			
	B	9	0.115	0.123	0.350			
	C	8	0.057	0.126	0.650			
	D	8	-0.095	0.127	0.450			
	E	5	0.081	0.129	0.530			
	F	3	Reference					
**Walking distance (km/day)**			0.035	0.016	0.029	-0.016	0.014	0.237
**Chain length (m)**			-0.032	0.013	0.013	-0.030	0.012	0.015
**Chain hour (h/day)**			0.005	0.003	0.093	0.009	0.003	0.008
**Roughage (kg/day)**			0.003	0.001	0.003	0.003	0.001	0.004
**Supplement (kg/day)**			0.021	0.003	<0.001	0.006	0.004	0.167

SE = Standard error

Variables having a P value <0.15 at the univariate analysis were included in the multivariate analysis.

Supplementary Table 4. Univariate and multivariate GEE analyses of demographic and camp management variables associated with 8-hydroxy-2' -deoxyguanosine concentrations.

Variable		N	Univariate analysis			Multivariate analysis		
			Estimate	SE	P value	Estimate	SE	P value
**Sex**								
	Male	14	Reference					
	Female	45	0.689	0.310	0.026	0.627	0.4167	0.133
**Age**			-0.002	0.013	0.860			
**Time**								
	T01	51	Reference					
	T02	53	1.038	0.918	0.258	1.044	0.872	0.231
	T03	50	0.304	0.468	0.516	0.184	0.450	0.683
	T04	46	0.018	0.465	0.970	-0.345	0.530	0.514
	T05	41	1.110	0.559	0.047	1.320	0.636	0.038
	T06	39	1.067	0.482	0.027	1.241	0.542	0.022
	T07	37	1.912	0.550	<0.001	1.556	0.569	0.006
**Camp**								
	A	13	-1.677	0.593	0.005	-2.067	0.663	0.002
	B	9	-2.643	0.557	<0.001	-1.818	0.725	0.012
	C	8	-2.694	0.557	<0.001	-2.022	0.613	0.001
	D	8	-0.736	1.056	0.486	-1.213	0.927	0.190
	E	5	-1.310	0.609	0.032	-1.804	0.804	0.025
	F	3	Reference					
**Walking distance (km/day)**			-0.306	0.092	<0.001	-0.257	0.181	0.156
**Chain length (m)**			0.165	0.105	0.120	0.296	0.137	0.030
**Chain hour (h/day)**			-0.049	0.023	0.035	-0.069	0.034	0.042
**Roughage (kg/day)**			-0.007	0.009	0.440			
**Supplement (kg/day)**			-0.029	0.034	0.380			

SE = Standard error
Variables having a P value <0.15 at the univariate analysis were included in the multivariate analysis.

Supplementary Table 5. Univariate and multivariate GEE analyses of demographic and camp management variables associated with heterophil counts.

Variable		N	Univariate analysis			Multivariate analysis		
			Estimate	SE	P value	Estimate	SE	P value
**Sex**								
	Male	14	Reference					
	Female	45	0.107	0.105	0.310			
**Age**			-0.005	0.004	0.230			
**Time**								
	T01	51	Reference					
	T02	53	-0.036	0.185	0.850			
	T03	50	-0.147	0.175	0.400			
	T04	46	-0.125	0.191	0.510			
	T05	41	-0.176	0.210	0.400			
	T06	39	-0.268	0.200	0.180			
	T07	37	-0.171	0.207	0.410			
**Camp**								
	A	13	-0.184	0.276	0.510	-0.237	0.294	0.420
	B	9	-0.461	0.281	0.100	-0.338	0.266	0.203
	C	8	-0.546	0.279	0.050	-0.537	0.272	0.051
	D	8	-0.210	0.296	0.480	0.094	0.274	0.732
	E	5	-0.078	0.332	0.810	-0.100	0.349	0.775
	F	3	Reference					
**Walking distance (km/day)**			-0.041	0.027	0.130	0.011	0.041	0.788
**Chain length (m)**			0.006	0.032	0.850			
**Chain hour (h/day)**			-0.007	0.007	0.280			
**Roughage (kg/day)**			0.005	0.002	0.036	0.007	0.003	0.021
**Supplement (kg/day)**			0.000	0.006	0.950			

SE = Standard error
Variables having a P value <0.15 at the univariate analysis were included in the multivariate analysis.

Supplementary Table 6. Univariate and multivariate GEE analyses of demographic and camp management variables associated with monocyte counts.

**Variable**		**N**	**Univariate analysis**			**Multivariate analysis**		
			**Estimate**	**SE**	**P value**	**Estimate**	**SE**	**P value**
**Sex**								
	Male	14	Reference					
	Female	45	0.124	0.215	0.560			
**Age**			-0.019	0.008	0.016	-0.017	0.007	0.026
**Time**								
	T01	51	Reference					
	T02	53	-0.434	0.242	0.073	-0.440	0.226	0.051
	T03	50	0.235	0.258	0.361	0.402	0.346	0.245
	T04	46	0.102	0.248	0.680	0.262	0.394	0.507
	T05	41	0.239	0.274	0.383	0.380	0.377	0.314
	T06	39	0.512	0.318	0.107	0.596	0.392	0.128
	T07	37	0.555	0.338	0.100	0.651	0.399	0.102
**Camp**								
	A	13	0.745	0.262	0.005	0.539	0.269	0.045
	B	9	-0.021	0.246	0.931	-0.153	0.270	0.569
	C	8	0.418	0.260	0.107	0.313	0.262	0.232
	D	8	0.485	0.260	0.062	0.307	0.305	0.313
	E	5	0.601	0.339	0.076	0.489	0.371	0.188
	F	3	Reference					
**Walking distance (km/day)**			-0.157	0.039	<0.001	-0.027	0.070	0.705
**Chain length (m)**			0.007	0.055	0.900			
**Chain hour (h/day)**			-0.009	0.007	0.200			
**Roughage (kg/day)**			-0.001	0.003	0.800			
**Supplement (kg/day)**			-0.025	0.010	0.009	0.018	0.019	0.363

SE = Standard error

Variables having a P value <0.15 at the univariate analysis were included in the multivariate analysis.

Supplementary Table 7. Univariate and multivariate GEE analyses of demographic and camp management variables associated with lymphocyte counts.

Variable		N	Univariate analysis			Multivariate analysis		
			Estimate	SE	P value	Estimate	SE	P value
**Sex**								
	Male	14	Reference					
	Female	45	-0.358	0.201	0.075	0.013	0.168	0.937
**Age**			-0.032	0.007	<0.001	-0.036	0.006	<0.001
**Time**								
	T01	51	Reference					
	T02	53	0.260	0.226	0.248	0.244	0.204	0.231
	T03	50	-0.069	0.217	0.752	0.117	0.3023	0.700
	T04	46	-0.933	0.217	<0.001	-0.818	0.347	0.018
	T05	41	-0.947	0.266	<0.001	-1.110	0.345	0.001
	T06	39	-0.770	0.303	0.011	-0.402	0.570	0.481
	T07	37	-1.540	0.258	<0.001	-0.767	0.643	0.233
**Camp**								
	A	13	0.080	0.323	0.806	-0.538	0.313	0.086
	B	9	0.320	0.327	0.328	0.209	0.541	0.700
	C	8	0.864	0.340	0.011	0.338	0.326	0.299
	D	8	0.172	0.352	0.624	0.269	0.473	0.569
	E	5	-0.242	0.364	0.506	0.269	0.446	0.546
	F	3	Reference					
**Walking distance (km/day)**			0.148	0.039	<0.001	-0.024	0.072	0.736
**Chain length (m)**			0.008	0.049	0.880			
**Chain hour (h/day)**			0.016	0.009	0.077	0.031	0.012	0.011
**Roughage (kg/day)**			0.007	0.003	0.011	0.006	0.004	0.120
**Supplement (kg/day)**			0.039	0.009	<0.001	0.011	0.018	0.562

SE = Standard error

Variables having a P value <0.15 at the univariate analysis were included in the multivariate analysis.

Supplementary Table 8. Univariate and multivariate GEE analyses of demographic and camp management variables associated with eosinophil counts.

Variable		N	Univariate analysis			Multivariate analysis		
			Estimate	SE	P value	Estimate	SE	P value
**Sex**								
	Male	14	Reference					
	Female	45	-0.102	0.053	0.054	-0.079	0.047	0.094
**Age**			-0.006	0.002	<0.001	-0.007	0.002	<0.001
**Time**								
	T01	51	Reference					
	T02	53	-0.016	0.066	0.810	-0.022	0.054	0.683
	T03	50	-0.088	0.058	0.130	-0.164	0.082	0.045
	T04	46	-0.090	0.065	0.170	-0.225	0.099	0.023
	T05	41	-0.052	0.069	0.450	-0.130	0.087	0.136
	T06	39	-0.087	0.070	0.220	-0.158	0.108	0.144
	T07	37	-0.040	0.064	0.540	-0.087	0.128	0.493
**Camp**								
	A	13	-0.126	0.122	0.301	-0.258	0.112	0.022
	B	9	-0.325	0.119	0.006	-0.242	0.126	0.055
	C	8	-0.428	0.118	<0.001	-0.493	0.113	<0.001
	D	8	-0.333	0.119	0.005	-0.225	0.123	0.067
	E	5	-0.219	0.125	0.079	-0.212	0.129	0.100
	F	3	Reference					
**Walking distance (km/day)**			-0.013	0.008	0.097	-0.010	0.015	0.533
**Chain length (m)**			0.034	0.010	<0.001	-0.011	0.012	0.384
**Chain hour (h/day)**			-0.004	0.001	0.009	-0.001	0.002	0.691
**Roughage (kg/day)**			0.003	0.001	<0.001	0.003	0.001	0.003
**Supplement (kg/day)**			-0.004	0.002	0.069	-0.009	0.005	0.067

SE = Standard error

Variables having a P value <0.15 at the univariate analysis were included in the multivariate analysis.

Supplementary Table 9. Univariate and multivariate GEE analyses of demographic and camp management variables associated with the heterophil/lymphocyte ratio.

Variable		N	Univariate analysis			Multivariate analysis		
			Estimate	SE	P value	Estimate	SE	P value
**Sex**								
	Male	14	Reference					
	Female	45	0.069	0.045	0.130	0.015	0.042	0.710
**Age**			0.004	0.002	0.019	0.004	0.002	0.012
**Time**								
	T01	51	Reference					
	T02	53	0.011	0.050	0.829	0.015	0.048	0.757
	T03	50	0.043	0.057	0.446	-0.010	0.073	0.891
	T04	46	0.070	0.058	0.224	0.039	0.089	0.657
	T05	41	0.113	0.066	0.086	0.143	0.082	0.052
	T06	39	0.132	0.076	0.081	0.057	0.178	0.749
	T07	37	0.308	0.081	<0.001	0.154	0.197	0.435
**Camp**								
	A	13	0.011	0.079	0.885	0.153	0.082	0.053
	B	9	-0.112	0.078	0.152	-0.099	0.15596	0.526
	C	8	-0.227	0.076	0.003	-0.126	0.08253	0.127
	D	8	-0.011	0.093	0.906	0.104	0.12294	0.398
	E	5	0.108	0.094	0.251	0.105	0.13501	0.435
	F	3	Reference					
**Walking distance (km/day)**			-0.023	0.011	0.039	0.042	0.018	0.021
**Chain length (m)**			-0.001	0.012	0.940			
**Chain hour (h/day)**			-0.006	0.002	0.014	-0.005	0.004	0.234
**Roughage (kg/day)**			-0.000	0.001	0.830			
**Supplement (kg/day)**			-0.006	0.002	0.010	-0.006	0.005	0.169

SE = Standard error

Variables having a P value <0.15 at the univariate analysis were included in the multivariate analysis.

Supplementary Table 10. Seasonal effects (mean ± SEM, range) on biomarkers of adrenal activity, oxidative stress, DNA damage, stress leukogram, and the heterophil/lymphocyte ratio in captive Asian elephants (n = 58) in six Thailand tourist camps during the COVID-19 pandemic and international travel ban between April 2020 – April 2022.

**Parameters**	**Summer**	**Rainy**	**Winter**
Fecal glucocorticoid metabolites (ng/g)	47.8 ± 1.4^{a} (30.8-79.8)	60.2 ± 2.1^{b} (38.0-90.2)	50.2 ± 2.3^{a} (26.5-103.5)
Malondialdehyde (µM)	2.3 ± 0.1^{a} (1.5-3.8)	2.2 ± 0.1^{a} (1.7-3.2)	2.2 ± 0.1^{a} (1.6-2.9)
8-hydroxy-2'-deoxyguanosine (ng/ml)	7.4 ± 0.4^{a} (4.7-14.3)	7.5 ± 0.4^{a} (4.3-17.2)	7.5 ± 0.4^{a} (4.5-15.8)
Heterophils (x 10^3 cells/µl)	3.1 ± 0.1^{a} (1.7-6.3)	3.0 ± 0.1^{a} (1.7-5.6)	2.8 ± 0.1^{a} (1.7-5.4)
Monocytes (x 10^3 cells/µl)	3.7 ± 0.2^{a} (1.6-6.4)	3.4 ± 0.1^{a} (2.1-5.7)	3.9 ± 0.2^{a} (2.2-7.2)
Lymphocytes (x 10^3 cells/µl)	3.9 ± 0.1^{a} (2.2-6.2)	4.4 ± 0.2^{a} (2.7-6.8)	4.3 ± 0.2^{a} (2.5-7.3)
Eosinophils (x 10^3 cells/µl)	0.3 ± 0.0^{a} (0.0-1.3)	0.4 ± 0.1^{a} (0.0-1.5)	0.3 ± 0.0^{a} (0.0-1.1)
Heterophil/Lymphocyte ratio	0.8 ± 0.0^{a} (0.4-2.1)	0.8 ± 0.0^{a} (0.3-1.7)	0.8 ± 0.1^{a} (0.3-1.6)

^{a,b,c}Values differ significantly across seasons ($P < 0.001$).
